# Simplified PCR-Based Quantification of Proteins with DNA Aptamers and Methylcellulose as a Blocking Agent

**DOI:** 10.3390/ijms25010347

**Published:** 2023-12-26

**Authors:** Oleksij Redcenko, Magda Tumova, Petr Draber

**Affiliations:** Laboratory of Signal Transduction, Institute of Molecular Genetics of the Czech Academy of Sciences, Vídeňská 1083, 142 20 Prague, Czech Republic; oleksij.redcenko@img.cas.cz (O.R.); magda.tumova@img.cas.cz (M.T.)

**Keywords:** DNA aptamers, polymerase chain reaction, polypropylene wells, immunoglobulins, blocking agents, methylcellulose

## Abstract

Due to their unique three-dimensional structure, DNA or RNA oligonucleotide aptamers bind to various molecules with high affinity and specificity. Aptamers, alone or in combination with antibodies, can be used to sensitively quantify target molecules by quantitative real-time polymerase chain reaction (qPCR). However, the assays are often complicated and unreliable. In this study, we explored the feasibility of performing the entire assay on wells of routinely used polypropylene PCR plates. We found that polypropylene wells efficiently bind proteins. This allows the entire assay to be run in a single well. To minimize nonspecific binding of the assay components to the polypropylene wells, we tested various blocking agents and identified methylcellulose as an effective alternative to the commonly used BSA. Methylcellulose not only demonstrates comparable or superior blocking capabilities but also offers the advantage of a well-defined composition and non-animal origin. Our findings support the utilization of aptamers, either alone or in combination with antibodies, for sensitive quantification of selected molecules immobilized in polypropylene PCR wells in a streamlined one-well qPCR assay under well-defined conditions.

## 1. Introduction

DNA and RNA aptamers represent a versatile class of synthetic single-stranded (ss) oligonucleotides known for their unique ability to adopt various three-dimensional conformations, enabling them to selectively bind to a wide array of molecular targets, including proteins, lipids, carbohydrates, and numerous small molecules. The isolation of aptamers typically involves an intricate process known as the systematic evolution of ligands by exponential enrichment (SELEX) [1,2,3]. SELEX relies on generating large libraries of random ssDNA or ssRNA oligonucleotides, followed by a series of selection rounds to enrich for sequences that exhibit high affinity and specificity for the desired target. Due to the increased interest in simplified aptamer production, several approaches have been developed into single-step protocols [4,5,6,7,8].

As a class of high-affinity ligands, oligonucleotide aptamers have several advantages over widely used antibodies, including synthetic production, longer shelf life, the flexibility to easily incorporate chemical modifications for labeling, and improved stability [3,9,10]. Aptamers are used as diagnostic tools ranging from simple enzyme-linked immunosorbent assays (ELISA), where the aptamer is used to immobilize the target molecule, which is subsequently detected by the antibody recognizing the target molecule. In these assays, the antibodies are conjugated to an enzyme (e.g., horseradish peroxidase, HRP) detectable by an enzymatic assay. Alternatively, aptamers are used in more complex assays involving various biosensors [11,12] or high-sensitivity assays in which selected DNA aptamers are used to bind to immobilized target analytes and, after washing off the unbound aptamer, are quantified in a quantitative real-time polymerase chain reaction (qPCR) [13,14,15]. These assays are similar to immuno-PCR (iPCR) techniques, in which antibodies are conjugated to short DNA fragments amplified by PCR techniques [16,17,18,19]. Although problems with the conjugation of antibodies and oligonucleotides have been partially overcome by the introduction of avidin or streptavidin and biotinylated DNA complexes [20,21,22] or by linking antibodies and DNA oligonucleotides via gold nanoparticles [23,24,25], the use of analyte-specific aptamers is a significant improvement, where aptamers serve to bind to immobilized targets and at the same time are used as DNA templates for amplification in qPCR. To simplify the assays, several modifications have been used, including the use of streptavidin-coated microwell strips and immobilization of the capture biotin-labeled aptamer followed by blocking the remaining binding sites, the addition of an analyte, and detection of the immobilized analyte by a reporter aptamer using qPCR [8].

In assays based on the quantification of an immobilized analyte, a nonspecific binding of antibodies, analyte, or aptamer during the assay can lead to erroneous results. To overcome the problem with nonspecific binding, the surface of microplate wells should be blocked with a blocking agent and then washed with detergent-containing wash buffer after each step of the assay. Commonly used blocking agents for polystyrene plates are BSA, skimmed milk casein, gelatin, and ovalbumin [26,27]. The selection of appropriate blocking agents generally depends on the solid support used, such as polystyrene or polypropylene, the type of specific reagents, such as antibodies or aptamers, and the source of the analyte [28]. The most commonly used blocking agent is BSA, which shows lot-to-lot variability reflecting the presence of various levels of contaminants such as bovine immunoglobulins and endogenous proteases. Furthermore, some blocking agents decrease the reactivity between the analyte-specific reagents and the analyte, possibly due to steric hindrance [29,30].

In our previous study, we found that methylcellulose, unlike carboxymethylcellulose, does not react with oligonucleotide aptamers and does not interfere with aptamer-analyte interactions [31]. In this study, we investigated whether methylcellulose could serve as a blocking agent in assays in which aptamers and antibodies are used as analyte-specific agents. Methylcellulose is a derivative of cellulose, where methyl groups substitute the hydroxyls at positions C-2, C-3, and/or C-6 in anhydro-D-glucose units [32]. Methylcellulose is a hydrophilic white powder that dissolves in cold water, which may be attributed to increased hydrogen bonding between the water molecules and oxygen in the remaining hydroxyl functional groups of the polymer [33].

We also studied whether widely used polypropylene strips and plates for qPCR could be used for immobilization of antibodies and aptamers, whether plates and strips for qPCR of different producers differ in their binding capacity for aptamers and antibodies, and whether the whole aptamer-based assays could be used in simplified and more sensitive tests. Our data indicate that analyte-specific aptamers alone or in combination with antibodies can be used to sensitively quantify selected molecules in a simple one-well qPCR assay under well-defined conditions.

## 2. Results

### 2.1. Binding of DNA Aptamers to Polypropylene Wells and Search for Agents Blocking This Binding

**Figure 1:**
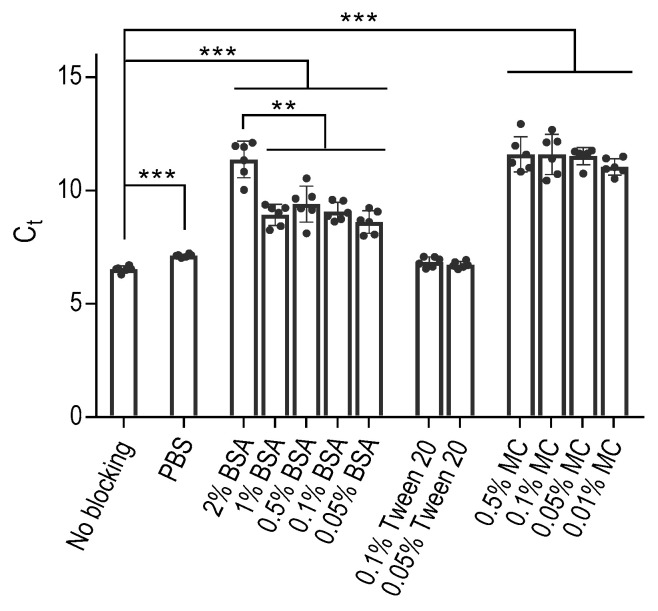
Aptamers bind to polypropylene PCR wells, as determined by qPCR, and this binding can be partially prevented by blocking the wells with BSA and even more so with methylcellulose (MC). The VWR polypropylene (PP) white 96-well plate wells were treated with the indicated concentrations of BSA, Tween 20, or MC in PBS, PBS alone, or untreated (no blocking). After 1 h at 37 °C, all wells were washed with PBS and incubated with 10 nM SP6 aptamer in PBS for 1 h at 37 °C. The wells were then washed with PBS to remove unbound aptamer, and the bound aptamer was quantified by qPCR using the aptamer-specific primers. Data represent means ± SD of a typical experiment conducted in hexaplicates from two similar experiments. Statistical significance of intergroup differences (unpaired Student’s *t*-test) is also indicated: ** *p* < 0.005, *** *p* < 0.0005.

In our initial experiments, we investigated the binding behavior of DNA aptamers to polypropylene PCR wells, mainly focusing on the effectiveness of BSA as a commonly used blocking agent to prevent this binding. To this end, we treated the wells of the 96-well PCR plate with various concentrations of BSA in PBS. Additionally, we included control wells that were either left untreated, treated with phosphate-buffered saline (PBS) alone, or PBS supplemented with Tween 20 at a final concentration of 0.1% (PBST) or 0.05%. Following a one-hour incubation, we carefully removed the blocking agents by washing the wells with PBS, after which we introduced an aptamer diluted in PBS to all wells. For this study, we used the ssDNA aptamer SP6, which is specific for the SARS-Cov-2 spike-binding protein; its sequence and origin are described in Table 1. One hour later, the unbound aptamer was removed by four consecutive washes with PBS. Subsequently, the presence of the bound aptamer was detected by qPCR after introducing into all wells a PCR mixture containing oligonucleotide primers designed for amplification of the aptamer in PCR. Our results showed that the polypropylene wells treated with PBS alone showed significantly (*p* < 0.0005) lower binding of the aptamer than untreated wells, as reflected by enhanced Ct values (Figure 1). The wells exposed to 0.1% or 0.05% Tween 20 showed similar cycle threshold (Ct) values to those treated with PBS or left untreated.

The use of BSA as a blocking agent significantly (*p* < 0.0005) reduced the aptamer binding. Decreasing concentrations of BSA from 2% to 1%, 0.5%, 0.1%, or 0.05% resulted in significantly (*p* < 0.005) higher aptamer binding to the polypropylene wells. Furthermore, we conducted experiments using methylcellulose as an alternative blocking agent, which does not interact with the aptamers [31]. Intriguingly, methylcellulose had a similar inhibitory effect on the aptamer binding to polypropylene surfaces as BSA at concentrations approximately one to two orders of magnitude lower. These results were consistent across additional experiments conducted with polypropylene wells from VWR and another aptamer with different specificity (Figure S1).

### 2.2. The ssDNA and Double-Stranded (dd)DNA Bind to Polypropylene Wells Comparably

**Figure 2:**
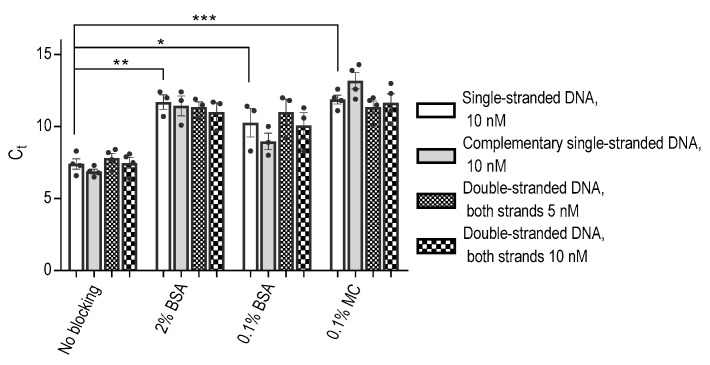
Various forms of DNA show similar binding to polypropylene wells, and the binding can be partially prevented by BSA and even more by MC. Aptamer SP6 and its complementary sequence were mixed, heated to 96 °C, and then cooled to 21 °C. Various forms of DNA were added at the indicated concentrations to the 96-well plate blocked with 2% BSA, 0.1% BSA, or 0.1% MC, as in Figure 1. The amount of the bound DNA was determined by qPCR using aptamer-specific primers. Data represent means ± SD of a typical experiment from three performed in triplicates or tetraplicates. Statistical significance of intergroup differences (unpaired Student’s *t*-test) is also indicated: * *p* < 0.05, ** *p* < 0.005, *** *p* < 0.0005.

The strong binding affinity observed between DNA aptamers and polypropylene wells presented an intriguing result, prompting us to delve deeper into the binding characteristics of ssDNA aptamers compared to their dsDNA counterparts. To investigate this, we conducted experiments involving the ssDNA aptamer and its corresponding ssDNA complementary strand, either separately or following their combination to form dsDNA. We found that both ssDNAs and dsDNAs bound to polypropylene wells similarly (Figure 2).

Furthermore, the inhibitory effect of BSA on the binding of both ssDNA and dsDNA to the polypropylene wells was found to be comparable. In agreement with the data in Figure 1, we found that methylcellulose at a concentration of 0.1% exhibited a more potent inhibitory effect on ssDNA than BSA at the same concentration. The same effect was observed for dsDNA (Figure 2). These observations provide valuable insights into the DNA binding to polypropylene wells and the differential inhibitory capabilities of methylcellulose and BSA on this binding.

### 2.3. Monoclonal Antibody (mAb) Binds to Polypropylene PCR Wells, and Methylcellulose Is a Good Blocker of Its Binding

Prior research has established the comparable binding properties of antibodies to some polypropylene and polystyrene well counterparts, with the additional insight that including PBST in the washing steps mitigates the nonspecific binding [34]. Building upon this knowledge, we sought to conduct a comprehensive assessment of polypropylene 96-well PCR plates from various manufacturers concerning antibody binding. Additionally, we investigated the inhibitory effects of the three blocking agents—BSA, Tween 20, and methylcellulose—in reducing nonspecific binding. We also included 96-well polystyrene plates commonly used in ELISA for comparative purposes.

**Figure 3:**
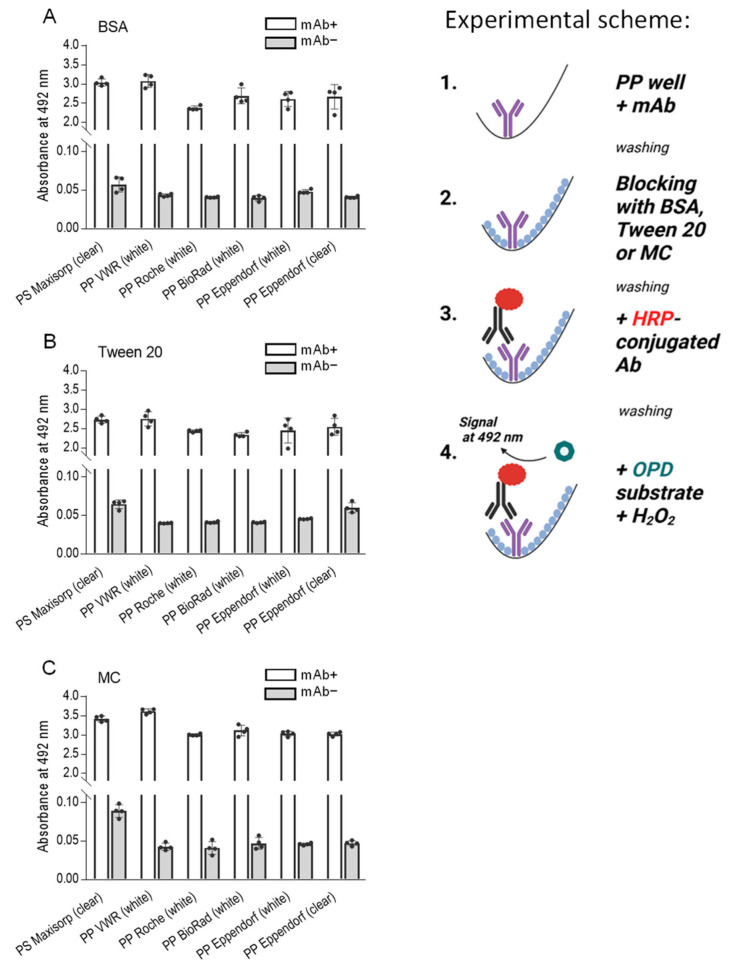
Methylcellulose (MC) is comparable to or better than BSA and Tween 20 as blocking agents in the ELISA assay used to detect the mAb bound to polypropylene surface. The experimental scheme of the experiments is shown on the right. The wells of a 96-well polystyrene (PS) plate (Nunc, Maxisorp), PP white plates (VWR, Roche, BioRad, Eppendorf), or PP transparent plates (Eppendorf) were filled with a mAb in PBS (0.5 µg/well, mAb+). PBS alone was used as a negative control (mAb−). After 1 h at 37 °C, the unbound mAb was washed out, and the wells were blocked for one hour at 37 °C with 0.1% BSA (**A**), 0.1% Tween 20 (**B**), or 0.1% MC (**C**) in PBS. After removal of the blocking agents, bound mAb was detected with HRP-conjugated anti-IgG antibodies. The binding of the HRP-labeled antibody was quantified spectrophotometrically after adding the HRP substrate, O-phenylenediamine (OPD), in the presence of hydrogen peroxide. Data represent means ± SD of a typical experiment from two performed in tetraplicate.

In these experiments, we introduced antibodies (mAb+) into the wells of the various plates and allowed them to incubate for one hour at 37 °C. Subsequently, unbound antibodies were meticulously washed. Control wells without antibodies (mAb−) served as a baseline for comparison. The binding sites remaining in the wells were blocked by 0.1% BSA (Figure 3A), 0.1% Tween 20 (Figure 3B), or 0.1% methylcellulose (Figure 3C). Bound antibodies were detected by an HRP-conjugated secondary antibody and an HRP substrate. Our findings revealed that the wells of polypropylene PCR plates from all manufacturers firmly bound the antibody. Furthermore, we observed that all blocking agents, in combination with washing steps using PBST, were effective in inhibiting the nonspecific binding of HRP-conjugated anti-IgG to polypropylene wells. Interestingly, nonspecific binding of HRP-labeled secondary antibodies was significantly higher in polystyrene plates blocked with methylcellulose than in all polypropylene plates (*p* < 0.0005). The best ratio between OD_492_ of positive (mAb+) and negative (mAb−) wells was observed in wells of VWR plates blocked with methylcellulose, reaching the value 84.8, calculated from the means of quadruplicates. When the same plates were blocked with BSA, or Tween 20, the ratios were 77.7 and 68.5, respectively. Interestingly, when ratios were calculated from polystyrene Maxisorp plates, the ratios for wells blocked with methylcellulose, BSA, or Tween 20 were 38.4, 53.0, and 42.3, respectively. Notably, the results obtained from polystyrene plates were similar to those of polypropylene plates, albeit with elevated levels of nonspecific binding in assays without mAb. These findings emphasize the relevance of the experimental platforms, including the selection of proper blocking agents and their concentrations.

We also performed a similar experiment in which we immobilized the nucleocapsid protein at concentrations 0–500 ng/well, blocked the remaining free binding sites with various blocking agents, and detected the bound nucleocapsid protein with an indirect ELISA using a mAb specific for the nucleocapsid protein, followed by an HRP-conjugated antibody developed as above. We found that nucleocapsid protein binds to polypropylene and polystyrene surfaces and that methylcellulose is comparable to BSA or Tween 20 used as blocking agents. This was reflected by the relatively low absorbance in control wells without nucleocapsid protein. Polystyrene wells in Maxisorp plates and polypropylene wells in VWR plates showed better binding of nucleocapsid, especially at low amounts of the nucleocapsid protein (15 and 7.5 ng; Figure S2).

### 2.4. Methylcellulose Efficiently Blocks the Nonspecific Binding of DNA Aptamers Used to Detect Target Proteins in Polypropylene Wells for PCR

**Figure 4:**
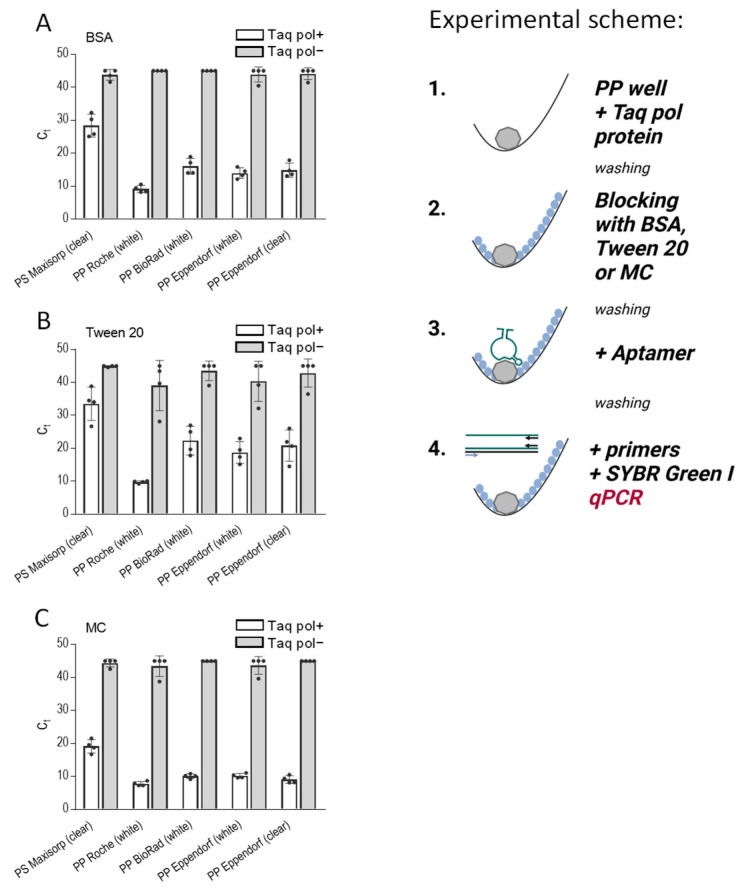
Methylcellulose efficiently blocks nonspecific binding in assays in which the analyte is immobilized in the PP wells and detected by the analyte-specific aptamer in qPCR. The scheme of the experiments is shown on the right. The wells of 96-well PS plates (Nunc, Maxisorp), white PP plates (Roche, BioRad, Eppendorf), and transparent PP plates (Eppendorf) were filled with PBS containing Taq DNA polymerase (0.5 µg/well; Taq pol+). PBS alone (Taq pol−) was used as a negative control. After 1 h at 37 °C, the unbound enzyme was washed out, and the wells were blocked with 0.1% BSA (**A**), 0.1% Tween 20 (**B**), or 0.1% MC (**C**) in PBS for one hour at 37 °C. After removing the blocking agents, anti-Taq pol-specific aptamer 44/70 in PBS-01% Tween 20 was added to all wells. After one hour, the unbound aptamer was washed out with PBST, and a qPCR master mix was added to amplify the bound aptamer. Data represent mean values ± SD of a typical experiment from three performed in tetraplicate.

In our subsequent experiment, we focused on the detection of the immobilized protein on various polypropylene 96-well PCR plates by aptamers. For comparison purposes, we also used a polystyrene ELISA plate. Our objective was to assess the effectiveness of three blocking agents, namely BSA (Figure 4A), Tween 20 (Figure 4B), and methylcellulose (Figure 4C), in preventing nonspecific binding of the DNA aptamer. For these experiments, we used as an analyte Taq DNA polymerase immobilized in the wells and anti-Taq aptamer 44/70. For all washing steps, we used PBST. For reference purposes, we included wells lacking Taq polymerase but subjected to the same treatment as the wells with the enzyme.

The data presented in Figure 4 underscore the superior performance of methylcellulose as a blocking agent, allowing efficient binding of the aptamer to the immobilized protein and, at the same time, preventing its nonspecific binding. Thus, in polypropylene plates from BioRad, Eppendorf (white), and Eppendorf (clear), Taq-specific aptamer binding to immobilized Taq DNA polymerase was significantly higher in methylcellulose-treated wells when compared to the wells blocked with BSA (*p* < 0.005). A significantly increased binding of the aptamer was also observed in Roche, BioRad, Eppendorf (white), and Eppendorf (clear) plates pretreated with methylcellulose compared to the plates with Tween 20-pretreated wells (*p* < 0.005). Interestingly, polystyrene Maxisorp plates exhibited lower binding of the aptamer to the immobilized enzyme than polypropylene plates, but again, the binding was significantly higher in plates pretreated with methylcellulose compared to BSA-pretreated cells (*p* < 0.005). In all plates tested, except Roche, the difference between positive wells (Taq pol+) and negative wells (Taq pol−) was higher in methylcellulose-treated than BSA- or Tween 20-treated wells.

### 2.5. Aptamer-Based iPCR (A-iPCR) with Methylcellulose as a Blocking Agent Is a Sensitive One-Well Assay for Detecting Low Concentrations of an Analyte

**Figure 5:**
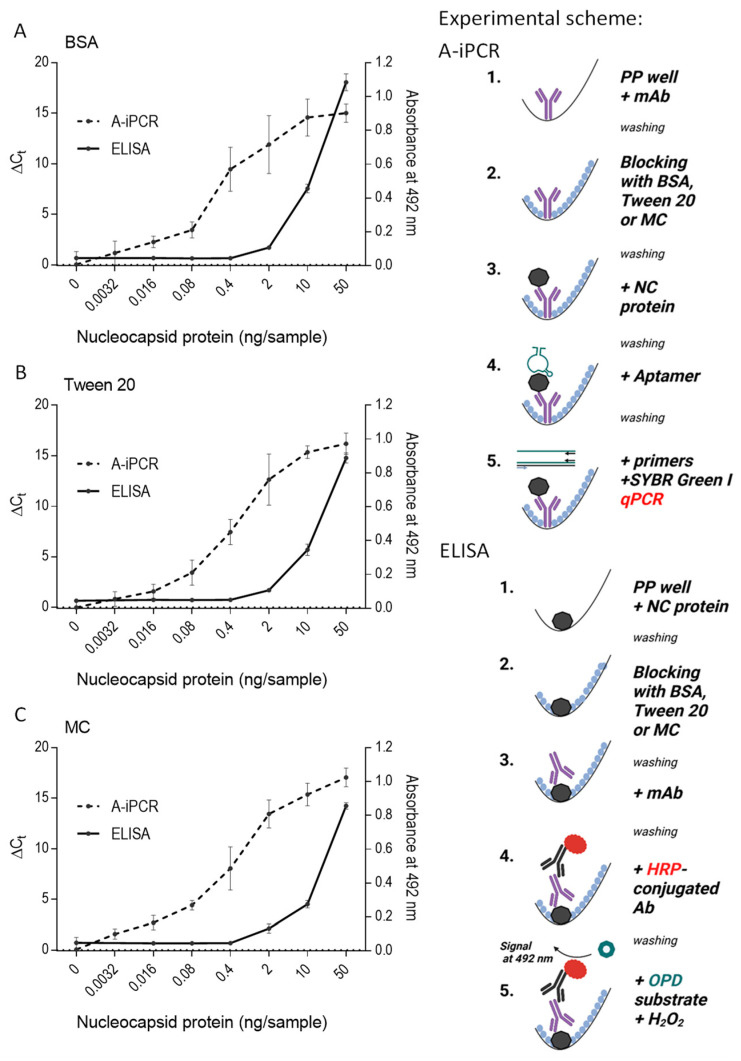
A-iPCR with methylcellulose as a blocking agent is a sensitive one-well assay for detecting low concentrations of an analyte. The schemes of the experiments are shown on the right. A mAb specific for the nucleocapsid protein was immobilized on the surface of wells of the polypropylene 96-well PCR plate. After removing unbound mAb and blocking free binding sites with 0.1% BSA (**A**), 0.1% Tween 20 (**B**), or 0.1% MC (**C**) in PBS, different amounts of nucleocapsid protein were introduced into the wells. The unbound protein was washed out with PBST in all wells. The bound protein was detected using nucleocapsid protein-specific aptamer and qPCR. ΔCt indicates the difference between amplification in the absence and presence of the nucleocapsid protein. The nucleocapsid protein was also quantified by direct ELISA, in which various amounts of the nucleocapsid protein were added to the wells of the Maxisorp polystyrene plate. Unbound protein was washed out, and the remaining binding sites were blocked, as described above. The bound protein was detected via the same nucleocapsid protein-specific mAb as used in A-iPCR, followed by HRP-conjugated secondary Ab and OPD/H_2_O_2_ substrate. Absorbance was measured at 492 nm. Data represent the mean values ± SD of a typical experiment from two performed in tetraplicate.

Aptamers frequently exhibit distinct binding sites in comparison to those recognized by antibodies, underscoring the significance of aptamers as target-specific tools in A-iPCR. To assess the efficacy of various blocking agents in A-iPCR, we designed a system where nucleocapsid-specific mAb was immobilized onto the wells of polypropylene 96-well plates. The plates were subsequently subjected to blocking by BSA (Figure 5A), Tween-20 (Figure 5B), or methylcellulose (Figure 5C). In this experimental setup, we introduced various concentrations of the nucleocapsid protein to the wells of the plate. Antibody-immobilized nucleocapsid protein was determined after adding a nucleocapsid-specific aptamer and quantifying its amount bound to wells by qPCR. The assay results were expressed as a difference (ΔCt) between Ct values obtained in the absence and presence of the nucleocapsid protein. Our findings demonstrated that methylcellulose performed comparably, and in some cases, even better than BSA or Tween-20, in preventing nonspecific absorption of the aptamer. Thus, when the nucleocapsid protein was used at a concentration of 0.0032 ng/sample, blocking free binding sites with methylcellulose resulted in a significant increase (*p* < 0.05) in ΔCt signal, whereas blocking with BSA or Tween 20 gave no significant change in ΔC_t_. This observation underscores the utility of methylcellulose as a promising blocking agent in the A-iPCR assays involving mAbs immobilized on polypropylene PCR surfaces.

The results obtained from the A-iPCR assay were compared with those of an ELISA, where the nucleocapsid protein at various concentrations was directly immobilized within the wells of Maxisorp polystyrene plates. Any unbound material was washed out, and the residual binding sites were subsequently blocked, employing the same methodology as described above. The nucleocapsid protein bound to the wells was detected with the same mAb as in the A-iPCR setup. The mAb bound to the nucleocapsid protein was detected by an HRP-conjugated anti-IgG antibody and an OPD/H_2_O_2_ substrate. The data presented in Figure 5A–C highlight that the direct ELISA assay exhibits lower sensitivity than the A-iPCR method; ELISA detected the nucleocapsid protein at an initial concentration of 2 ng/sample, whereas A-iPCR detected it at a concentration of 0.0032 ng/mL. In ELISA, a similar sensitivity of the assay was observed in wells blocked with BSA, Tween 20, or MC. These findings underscore the significance of methylcellulose as an effective blocking agent.

## 3. Discussion

Aptamers offer substantial promise as ligands for detecting various analytes due to their remarkable specificity, ease of preparation, stability, and ability to be easily detected in commonly used PCR techniques. Simple single-tube assays utilizing aptamers could detect immobilized analytes within PCR plate wells through the PCR process. In this study, we aimed to assess the feasibility of such simplified aptamer-based assays.

Initially, we investigated the binding of aptamers to the polypropylene wells of the PCR plates. Our data indicated that the aptamers in PBS bind strongly to the polypropylene wells, similar to their binding to polystyrene wells. This robust binding was observed across various aptamers, suggesting that the analyte specificity of the aptamers does not primarily influence the binding. This conclusion was supported by our finding that both ssDNA aptamers, ssDNA oligonucleotides, and dsDNA oligonucleotides of the same length exhibited similar binding to propylene surfaces. These findings align with prior research demonstrating the analogous binding behavior of ssDNA and dsDNA to polypropylene surfaces [35,36].

**Figure 6:**
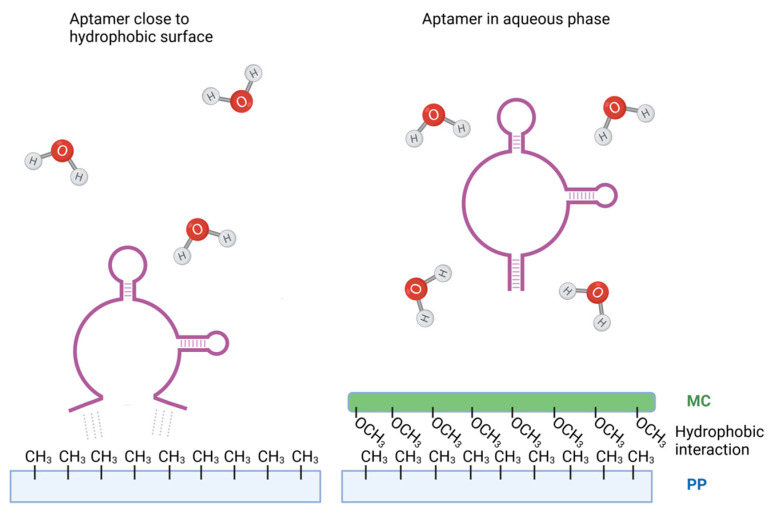
Model of possible binding of DNA aptamer to polypropylene surface and inhibition of the binding by methylcellulose. In the presence of polypropylene, hydrophobic nitrogen bases in DNA aptamers tend to interact with hydrophobic methyl groups of polypropylene to minimize contact with water. This could be the driving force behind the binding of DNA aptamers to polypropylene surfaces (**left**). When polypropylene surface is treated with methylcellulose, the methyl groups of methylcellulose react with methyl groups of polypropylene via van der Waals forces, reducing interactions of aptamers with the polypropylene surfaces (**right**). Aptamers surrounded by water molecules could have different conformations, explaining different properties of the soluble and immobilized aptamers.

The molecular mechanism behind DNA binding to polypropylene remains incompletely understood. DNA is a highly charged, hydrophilic molecule, whereas polypropylene, used in most microtubes and microplates for PCR, is very hydrophobic. These characteristics should reduce the interaction of DNA with polypropylene surfaces. Interestingly, Gaillard and colleagues demonstrated that DNA binding to polypropylene surfaces increases at higher ionic strength conditions [37]. It is likely that higher ionic strength can shield the negatively charged regions of DNA, promoting hydrophobic interactions. Additional experiments suggested that the hydrophobic DNA nitrogen bases tend to cluster within the dsDNA molecule in a hydrophilic environment to minimize contact with water and expose the negatively charged phosphate backbone on the surface. However, in a hydrophobic environment, this promotes base unstacking [38,39], possibly explaining the mechanism of dsDNA binding to polypropylene and other hydrophobic plastic. Since ssDNA aptamers expose nitrogen bases, form secondary structures through base pairing, and define target affinities through tertiary structures, a similar mechanism may underlie the interactions between aptamers and polypropylene (Figure 6). Van der Waals forces primarily drive adsorption forces, contributing to the interaction of aptamers with hydrophobic surfaces.

Subsequently, we explored the potential of a widely used blocker of nonspecific binding, BSA, in inhibiting aptamer binding to the wells of polypropylene PCR plates. As expected, pretreatment of the wells with BSA led to reduced aptamer binding, depending on the BSA concentration. In these studies, we also included methylcellulose as a blocking reagent, which we have previously shown not to react with aptamers [31]. Surprisingly, methylcellulose exhibited a more substantial inhibitory effect on aptamer binding than BSA. Methylcellulose, a non-ionic polymer with amphiphilic properties, binds to plastic through hydrophobic interactions between the methyl groups on the polypropylene surface and methylcellulose. In aqueous solutions and in the absence of polypropylene, the aqueous phase shields the hydrophobic methyl groups of methylcellulose [40].

Methylcellulose stands out as an advantageous blocking agent due to the thickness of the adsorbed layer and higher viscosity [32], which provides a more practical physical barrier against nonspecific binding. Additionally, compared to BSA, methylcellulose has a lower net surface charge, reducing the potential for electrostatic interactions with various analyte components, especially those with varying charges.

In the next set of experiments, we evaluated antibody binding to the polypropylene PCR wells and the effectiveness of BSA and methylcellulose in reducing the nonspecific binding. In these experiments, the antibodies in PBS were allowed to bind to polypropylene surfaces, and the remaining binding sites were blocked with BSA, Tween 20, or methylcellulose. All subsequent washing steps after treatment with the blocking agents were done with PBST to further reduce the nonspecific binding of the secondary HRP-labeled antibody. The results showed that methylcellulose was comparable to or superior to BSA in blocking antibody binding. Pretreatment of the polypropylene wells with PBST did not reduce the binding of antibodies to the wells. Nevertheless, including Tween 20 in the washing buffer reduced nonspecific binding as described in previous studies [34,41,42,43]. Based on these findings, we recommend a protocol that involves binding the analyte or analyte-specific antibodies in PBS into polypropylene PCR wells, followed by applying methylcellulose in PBS, with all subsequent washing steps carried out in PBST. This approach resulted in a low background signal and facilitated the quantification of aptamers by qPCR.

Collectively, our data support the notion that direct or indirect analyte immobilization within polypropylene PCR wells, in conjunction with methylcellulose as a blocking agent and the quantification of aptamers bound to the analyte through qPCR, presents a straightforward method for analyte quantification employing analyte-specific aptamers. Including methylcellulose instead of BSA in this assay reduces costs and eliminates the presence of animal contaminants, making it a promising choice for future applications.

## 4. Materials and Methods

### 4.1. Materials and Reagents

In this study, we used 96-well polypropylene microplates for PCR from different suppliers: Twin.tec^®^ PCR Plate, colorless (Eppendorf, Hamburg, Germany; Cat. No. 0030128648); Twin.tec^®^ 96 PCR Plate, white (Eppendorf; Cat. No. 0030132505); PCR white plates (VWR International, Radnor, PA, USA; Cat. No. 211-0313); LightCycler 480 Multiwell Plate 96-well, white (Roche Diagnostics, Mannheim, Germany; Cat. No. 04729692001); Multiplate™ 96-Well PCR Plates, white (BioRad, Hercules, CA, USA; Cat. No. MLL 9651). We also used 96-well Immuno polystyrene plates Maxisorp, flat-bottomed (Thermo Scientific Nunc, Roskilde, Denmark; Cat. No. 456537). HisPur cobalt resin (Cat. No. 89964) was purchased from Thermo Fisher Scientific (Rockford, IL, USA) and Streptavidin MagneSphere^®^ Paramagnetic Particles (Cat. No. Z5481) from Promega (Madison, WI, USA). Trehalose and propanediol-based PCR master mix (Cat. No. T601), DNA polymerase from *Thermus aquaticus* (Taq; Cat. No. T032), and PCR agarose (Cat. No. P045) were obtained from Top-Bio, Vestec, Czech Republic. Taq DNA polymerase used as an analyte was extensively dialyzed against PBS. Peroxidase-AffiniPure Goat Anti-Mouse IgG (H+L) secondary antibody was purchased from Jackson Immune Research (West Grove, PA, USA; Cat. No. 115-035-003). Absorbance was measured in polystyrene flat-bottom immuno nonsterile 96-well Maxisorp plates at 492 nm using a multimode microplate reader Tecan Infinite M200 (Tecan Trading AG, Grodig, Austria). PBST was prepared by mixing PBS with Tween 20 (Merck final concentration 0.1%). Protease-free grade BSA was obtained from VWR International (Cat. No 422361V), and methylcellulose was from Sigma Aldrich (Cat. No M0512). SARS-CoV-2 nucleocapsid protein HEK293 (Cat No. RD975598100) was obtained from BioVendor R&D (Brno, Czech Republic). Oligonucleotides, DNA aptamers and primers used for their PCR amplification were obtained from Sigma-Aldrich (Merck; Darmsta); their composition is described in Table 1.

The origin and properties of the SP6 aptamer have been described [44]. A mAb specific for Taq DNA polymerase (anti-Taq) was prepared as previously described [45]. Anti-nucleocapsid mAb was prepared by immunization of BALB/c mice with SARS-CoV-2 nucleocapsid protein and hybridoma technology in the mAb Service Laboratory at the Institute of Molecular Genetics, Prague, Czech Republic. All other chemicals were from Sigma-Aldrich (Merck).

### 4.2. Isolation of the Aptamer 44/70, Specific for Taq DNA Polymerase

DNA aptamer 44/70 specific for Taq DNA polymerase was prepared from a ssDNA library containing a 30-nucleotide random oligonucleotide sequence (30N) with two constant PCR primer regions: CCTTGAACCTGTGCCATTTG-30N-GAACAGTAGGAAGATGGAGG. Aptamer selection was performed in two stages. Each stage consisted of several rounds. In the first stage, a random ssDNA library in PBST was incubated with the recombinant Taq DNA polymerase immobilized in cobalt resin. After a 30 min incubation, unbound oligonucleotides were washed with PBST and finally with water. Then, the resin pool was distributed among PCR wells, and the oligonucleotides bound to the resin were amplified by PCR. The PCR dsDNA amplicons were transformed into ssDNA aptamers by asymmetrical PCR with a biotin-labeled reverse primer. The PCR amplicons were precipitated with ethanol/sodium acetate. The precipitates were solubilized with PBST, incubated with streptavidin-coated magnetic beads, and the dsDNA with a biotin-labeled reverse strand was removed by magnetic separation. Such a selection step was repeated five times. In the second stage, an anti-Taq mAb in PBS was immobilized on the surface of the polypropylene PCR wells. After 1 h at 37 °C, the wells were washed and treated for 1 h at 37 °C with 0.1% methylcellulose in PBS to block the remaining binding sites. Taq DNA polymerase in PBST was added to the wells and incubated for 1 h at 37 °C. Then, the wells were washed with PBST to remove the unbound enzyme. The aptamers preselected in the first stage were added to the wells, and the plates were incubated at 37 °C. After 1 h, the wells were washed five times, a PCR mixture with the corresponding primers was added, and the aptamers were amplified by PCR. Before each round, a negative selection was made in which oligonucleotides were incubated in wells treated and processed as above, except that the antigen was not bound to antibodies. The PCR conditions for dsDNA production after positive selection were as follows: 5 min at 95 °C, followed by 15 s at 95 °C, 5 s at 57 °C, and 7 s at 72 °C (30 cycles). For ssDNA production, the conditions were: 5 min at 95 °C, followed by 10 s at 95 °C, 3 sec at 96 °C, 3 s at 58 °C, and 5 s at 72 °C (45 cycles). After PCR, the quality of the DNA amplicons was monitored by electrophoresis on a 2% agarose gel. For cloning the PCR amplicons, the Qiagen PCR cloning kit (Qiagen, Hilden, Germany; Cat. No. 231124) was used as recommended by the manufacturer. dsDNA was incubated for 10 min at 72 °C (to get TA overhang) and then ligated into the pDrive cloning vector. The ligated mixture was transformed into QIAGEN EZ-competent cells, and the grown colonies were picked up and incubated in LB media at 37 °C. Bacterial cultures were diluted 50 times in water, and 1 µL aliquots were used as templates in PCR. SEQme company (Dobris, Czech Republic) performed DNA sequencing.

### 4.3. Nonspecific Binding of Aptamers to the Wells of Polypropylene PCR Plates

The wells of a 96-well polypropylene plate (VWR, white) were filled with 300 µL of PBS alone or supplemented with various concentrations of BSA, Tween 20, and methylcellulose. Some wells were left empty. After 1 h at 37 °C, the wells were washed 3× with PBS and incubated with 50 µL of 10 nM SP6 aptamer for 1 h at 37 °C. The unbound aptamer was washed out with PBS (5×). We used these mild washing conditions without Tween 20 to differentiate the potential distinct effects of the particular blocking agents. Then, 50 µL of qPCR Master Mix with SYBR Green I and aptamer-specific primers were dispensed into the wells. The bound aptamer was quantified by qPCR in a qTower3G cycler (Analytik Jena, Jena, Germany), which also automatically calculated Ct values using a qPCRsoft 4.0 touch software for qTower3G (Analytik Jena). The following cycling conditions were used: 4 min at 95 °C, followed by 45 cycles of 10 s at 95 °C, 7 s at 57 °C, and 14 s at 72 °C.

To check the binding properties of ssDNA and dsDNA, we used the SP6 aptamer and its complementary sequence. For the ssDNA test, 50 µL of 10 nM SP6 aptamer or the complementary sequence to SP6 aptamer were used. For the dsDNA test, 50 µL of a 5 nM or 10 nM mixture of both sense (SP6 aptamer) and anti-sense ssDNA was prepared. All DNA samples were denatured at 95 °C for 5 min and then cooled to RT.

### 4.4. Quantification of Proteins Directly Immobilized in the Polypropylene PCR Wells

We used Taq DNA polymerase in PBS and the corresponding 44/70 anti-Taq aptamer to develop the assay. We added 0.5 µg of the protein in 100 µL PBS per well of polypropylene plates for PCR. As a control, we also used conventional ELISA Maxisorp plates. Wells with PBS alone were used as negative controls. The plates were incubated for 1 h at 37 °C, followed by 2× washing with PBS. Then, the remaining binding sites were blocked with 0.1% BSA, 0.1% Tween 20, or 0.1% methylcellulose for 1 h at 37 °C or left unblocked in PBS, followed by 2× washing with PBS. The aptamers 44/70, specific to Taq DNA polymerase, at a concentration of 50 nM in PBST were then incubated in the wells for 1 h at 21 °C to bind to the target proteins. The unbound aptamers were washed out in five washing cycles with PBST; each washing cycle included shaking the plate for 30 s on the Tecan HydroSpeedTM plate washer (Tecan Trading AG, Switzerland). The PCR mixture containing primers for the aptamer (final concentration 300 nM) was added directly into all wells, and real-time PCR was run with Sybr Green I detection on a qTower3G Analytik Jena cycler (Analytik Jena, Jena, Germany). The following cycling conditions were used: 4 min at 95 °C, followed by 45 cycles of 10 s at 95 °C, 7 s at 55 °C, and 14 s at 72 °C. When the Maxisorp polystyrene plates were used, the plates were incubated with the PCR mixture at 95 °C for 5 min. Then, the samples were transferred into a new polypropylene PCR plate to quantify the aptamers by qPCR. All measurements were performed in triplicates, and average Ct was used to calculate ΔCt between positive and negative samples for each plate and particular condition.

### 4.5. A-iPCR

The sandwich A-iPCR binding assay in the wells of the polypropylene PCR plates was tested with anti-nucleocapsid aptamer. Anti-nucleocapsid mAb (0.15 µg per well) in 100 µL of PBS was distributed into wells of the PCR plates. After 1 h of incubation at 37 °C, the wells were washed two times with PBS (each wash consisted of incubation with PBS for 1 min at room temperature before PBS removal). PBS supplemented with 0.1% BSA, 0.1% Tween 20, or 0.1% methylcellulose was used to block the remaining free binding sites. After the blocking and washing steps, different amounts of the nucleocapsid protein (diluted in PBST) were added to the immobilized antibodies and incubated for 1 h at 37 °C, then washed two times with PBST (each time incubated for 1 min at 21 °C). The nucleocapsid-specific aptamer (final concentration 100 pM in PBST) was added to the wells and incubated for 1 h at 37 °C. After incubation with the aptamer, the wells were washed five times with PBST; each washing step lasted 5 min at 21 °C. Following the final washing step, the plate was centrifuged at 1500 rpm for 1 min, and the remaining solution was sucked off from the wells. The PCR mixture with the primers (300 nM) to amplify the aptamer was added directly to the wells, and qPCR was run using a qTower3G Analytik Jena cycler. The following PCR conditions were used: 4 min at 95 °C, followed by 10 s at 95 °C, 5 s at 57 °C, and 14 s at 72 °C (45 cycles). The fluorescent signal during qPCR corresponds to the number of aptamers bound to the protein immobilized in the wells by antibodies. For quantification of the binding assay result, we used the ΔCt value, calculated by subtracting the Ct value of each replicate from the average of the Ct values of negative samples (CtNS): ΔCt = CtNS (mean) − Ct (observed).

### 4.6. ELISA

Direct ELISA was carried out through direct adsorption of proteins (antigens or antibodies) on a solid surface in flat-bottom polystyrene Maxisorp clear 96-well plates or PCR polypropylene plates, as indicated in Results. The protein of interest was dissolved in PBS, and 100 µL aliquots were added to the wells. After incubation for 1 h at 37 °C, the wells were washed two times with PBS (each wash for 1 min at 21 °C). Then, the free binding sites were blocked at 37 °C with PBS supplemented with 0.1% BSA, 0.1% Tween 20, or 0.1% methylcellulose. After 60 min, the wells were washed two times with PBST 0.1%, and the mAb was added. After incubating for 1 h at 37 °C, the wells were washed two times with PBST (each wash for 1 min at 21 °C). HRP-labeled-AffiniPure goat anti-mouse IgG diluted in PBS 1:10,000 was then added, and the plate was incubated for 45 min at 37 °C. Then, the unbound antibody was removed by washing with PBST (5 times with PBST for 1 min at 21 °C). The HRP activity was determined using H_2_O_2_ and OPD as substrates [47,48]. The reaction was stopped by adding 1 M H_2_SO_4_. When PCR plates were used, the solution from each well was transferred to the flat-bottom polystyrene Maxisorp plate. The absorbance was measured at 492 nm using a TECAN plate reader, the Infinite M200.

## Figures and Tables

**Table 1:** DNA oligonucleotides and the corresponding forward primers.

SP6 aptamer *	GGGAGAGGAGGGAGATAGATATCAACCCATGGTAGGTATTGCTTGGTAGGGATAGTGGGCTTGATGTTTCGTGGATGCCACAGGAC
F primer	GGGAGAGGAGGGAGATAGATATCAA
R primer	GTCCTGTGGCATCCACGAAA
Complementary to SP6	GTC CTG TGG CAT CCA CGA AAC ATC AAG CCC ACT ATC CCT ACC AAG CAA TAC CTA CCA TGG GTT GAT ATC TAT CTC CCT CCT CTC CC
44/70 aptamer **	CCTTGAACCTGTGCCATTTGCTAATTGAGACTATTATGGGCTTTTTAGTCGAACAGTAGGAAGATGGAGG
F primer	CCTTGAACCTGTGCCATTTG
R primer	CCTCCATCTTCCTACTGTTC
NC aptamer ***	GCAATGGTACGGTACTTCCGGATGCGGAAACTGGCTAATTGGTGAGGCTGGGGCGGTCGTGCAGCAAAAGTGCACGCTACTTTGCTAA
F primer	GCAATGGTACGGTACTTCC
R primer	TTAGCAAAGTAGCGTGCACTTTTG

* SARS-CoV-2 spike binding aptamer [44] and the corresponding forward (F) and reverse (R) oligonucleotide primers. ** Taq DNA polymerase binding aptamer, prepared as described in Section 4.2, and the corresponding oligonucleotide primers. *** SARS-CoV-2 nucleocapsid binding aptamer and the corresponding oligonucleotide primers [46].

## Data Availability

All data are provided herein.

## Supplementary Materials

The following supporting information can be downloaded at: https://www.mdpi.com/article/10.3390/ijms25010347/s1.
