# Supplementary material for: Simplified PCR-Based Quantification of Proteins with DNA Aptamers and Methylcellulose as a Blocking Agent
Source: Int J Mol Sci. 2023 Dec 26;25(1):347. doi: 10.3390/ijms25010347 (PMC10779054; doi:10.3390/ijms25010347)
Supplement: Supplementary file 1 [file ijms-25-00347-s001.zip › ijms-2729170-supplementary.pdf]

Supplementary material to:

Simplified PCR-Based Quantification of Proteins with DNA Aptamers and Methylcellulose as a Blocking Agent

Oleksij Redcenko [1,†], Magda Tumova [1,†] and Petr Draber [1,*]

Laboratory of Signal Transduction, Institute of Molecular Genetics of the Czech Academy of Sciences, Víde ˇnská 1083, 142 20 Prague, Czech Republic; oleksij.redcenko@img.cas.cz (O.R.); magda.tumova@img.cas.cz (M.T.)
* Correspondence: draberpe@img.cas.cz
† These authors contributed equally to this work.

Contents

Figure S1. Methylcellulose inhibits the binding of 44/70 DNA aptamer and SP6 DNA aptamer to PCR wells similarly to or better than BSA.

Figure S2. A methylcellulose is comparable to BSA or Tween 20 as a blocking agent in the indirect ELISA to detect the nucleocapsid protein.

Figure S3. A methylcellulose-based blocking buffer, freshly prepared or stored for 120 days at 4 °C, is better than freshly prepared BSA-based blocking buffer in ELISA.

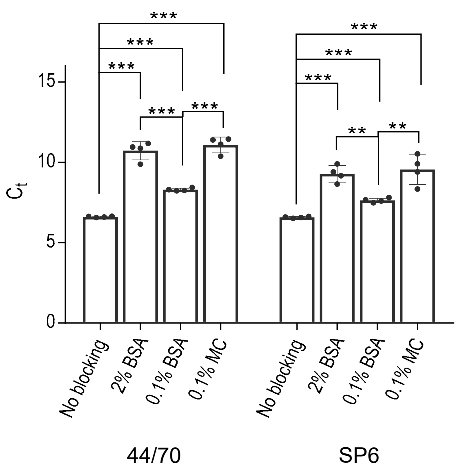

Figure S1. Methylcellulose inhibits the binding of 44/70 DNA aptamer and SP6 DNA aptamer to PCR wells similarly to or better than BSA. Wells of a VWR white 96-well PP plate were filled with MC or BSA at the indicated concentrations in PBS or with PBS alone as a control (No blocking). After 1 hour at 21 °C, all wells were washed with PBS and incubated with 10 nM 44/70 or SP6 aptamer in PBS for 1 hour at 37 °C. The wells were then washed with PBS to remove unbound aptamers, and the amounts of the aptamers bound were determined by qPCR using the aptamer-specific primers. Data represent means ± S.D. of an experiment conducted in tetraplicates. Statistical significance of intergroup differences (unpaired Student's *t*-test) are indicated: ** $P<0.005$ *** $P<0.0005$.

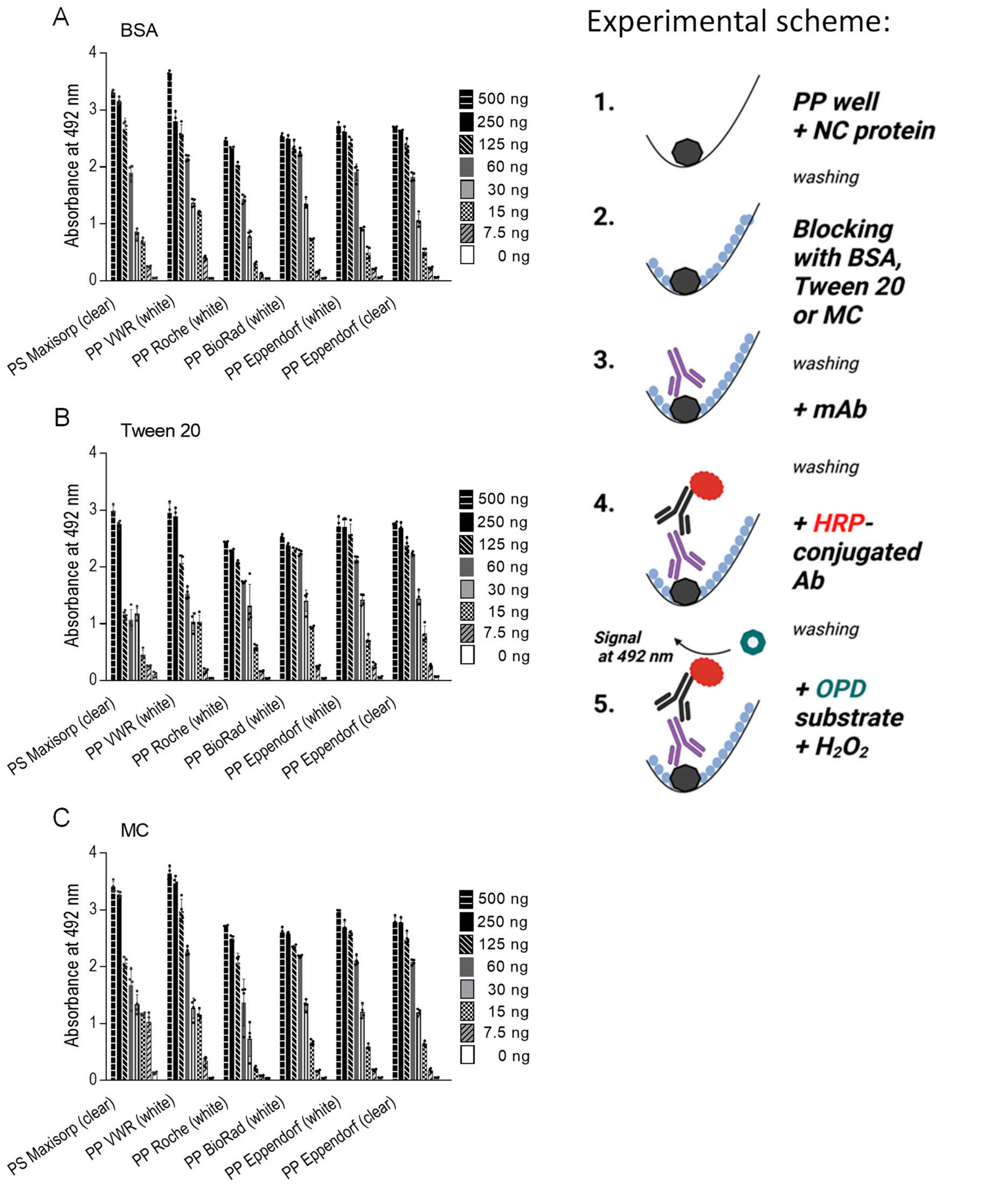

Figure S2. A methylcellulose is comparable to BSA or Tween 20 as a blocking agent in the indirect ELISA to detect the nucleocapsid protein. The wells of 96-well polystyrene (PS) plates (Nunc, Maxisorp), polypropylene (PP) white plates (VWR, Roche, BioRad, Eppendorf) or PP transparent plates (Eppendorf) were filled with various amounts of the nucleocapsid protein (0 – 500 ng/well) in PBS. After 1 hour at 37 °C, the unbound protein was washed out, and the wells were blocked for 1 hr at 37 °C with 0.1% BSA (**A**), 0.1% Tween 20 (**B**) or 0.1% MC (**C**); all in PBS. After removing unbound blocking reagents, the bound nucleocapsid protein was determined by anti-nucleocapsid mAb followed by HRP-conjugated anti-IgG antibody and OPD/H_2O_2 as a substrate. Data represent means ± S.D. from an experiment performed in tetraplicates.

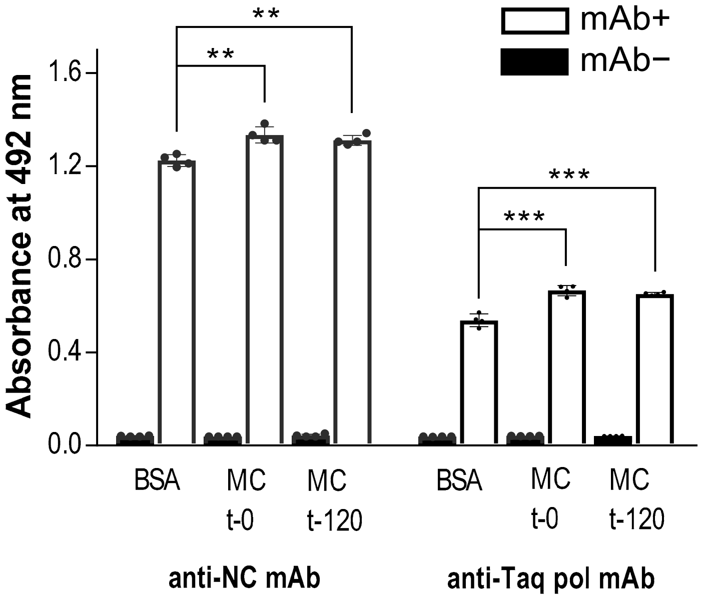

Figure S3. A methylcellulose-based blocking buffer, freshly prepared or stored for 120 days at 4 °C, is better than freshly prepared BSA-based blocking buffer in ELISA. Wells of 96-well polypropylene white plates (VWR) were filled with PBS containing a mAb (mAb+) specific for the nucleocapsid protein (anti-NC; 0.5 µg/well) or Taq DNA polymerase (anti-Taq pol; 0.5 µg/well). PBS alone was used as a negative control (mAb−). After 1 hour, the unbound mAbs were washed out, and the wells were blocked with freshly prepared 0.1% BSA in PBS or 0.1% MC in PBS prepared from 1% MC freshly prepared stock (t-0) or 1% MC stock stored for 120 days at 4 °C (t-120). After removal of the blocking agents, the bound mAbs were detected with HRP-conjugated anti-IgG antibody diluted 1:30 000. The binding of the HRP-labeled antibody was quantified spectrophotometrically after adding HRP substrate O-phenylenediamine and H_2O_2. Data represent means ± SD from a typical experiment out of three performed in tetraplicates. Statistical significance of intergroup differences (unpaired Student's t-test) is indicated: ** $P<0.005$; *** $P<0.0005$.
